# Clinical Evaluation of Extracellular ADMA Concentrations in Human Blood and Adipose Tissue

**DOI:** 10.3390/ijms15011189

**Published:** 2014-01-17

**Authors:** Marcus May, Sandor Batkai, Alexander A. Zörner, Dimitrios Tsikas, Jens Jordan, Stefan Engeli

**Affiliations:** 1 Institute of Clinical Pharmacology, Hannover Medical School, Carl-Neuberg-Straße 1, 30625 Hannover, Germany; E-Mails: may.marcus@mh-hannover.de (M.M.); Zoerner.Alexander@mh-hannover.de (A.A.Z.); Tsikas.Dimitrios@mh-hannover.de (D.T.); Jordan.Jens@mh-hannover.de (J.J.); 2 Institute of Molecular and Translational Therapeutic Strategies, Hannover Medical School, Carl-Neuberg-Straße 1, 30625 Hannover, Germany; E-Mail: Batkai.Sandor@mh-hannover.de

**Keywords:** asymmetric dimethylarginine, nitric oxide, nitric oxide synthase, dimethylarginine dimethylaminohydrolase, adipose tissue, diabetes, obesity

## Abstract

Circulating asymmetrical dimethylarginine (ADMA), an endogenous inhibitor of nitric oxide synthesis, has been proposed as a biomarker for clinical outcome. Dimethylarginine dimethylaminohydrolase (DDAH) is the main enzyme responsible for ADMA metabolism and elimination. Adipose tissue ADMA concentrations and DDAH activity and their role in diabetes and obesity have not yet been investigated. In this study, we evaluated clinical microdialysis in combination with a sensitive analytical method (GC-MS/MS) to measure ADMA concentrations in extracellular fluid. Adipose tissue ADMA concentrations were assessed before and during an oral glucose tolerance test in lean healthy subjects and subjects with diabetes (*n* = 4 each), and in morbidly obese subjects before and after weight loss of 30 kg (*n* = 7). DDAH activity was determined in subcutaneous and visceral adipose tissue obtained during laparoscopic surgery (*n* = 5 paired samples). Mean interstitial ADMA concentrations did not differ between study populations (healthy 0.17 ± 0.03 μM; diabetic 0.21 ± 0.03 μM; morbidly obese 0.16 ± 0.01 and 0.17 ± 0.01 μM before and after weight loss, respectively). We did not observe any response of interstitial ADMA concentrations to the oral glucose challenge. Adipose tissue DDAH activity was negligible compared to liver tissue. Thus, adipose tissue ADMA plays a minor role in NO-dependent regulation of adipose tissue blood flow and metabolism.

## 1. Introduction

Endothelial function is already impaired in obesity and insulin resistance [1,2] and further deteriorates with the development of type 2 diabetes and cardiovascular disease [3,4]. Adipose tissue-derived metabolites, hormones and cytokines may play a major pathophysiological role in obesity-associated endothelial dysfunction [5–7]. One of the main candidate molecules for the link between endothelial dysfunction, cardiovascular disease, and insulin resistance is the endogenous nitric oxide synthase (NOS) inhibitor asymmetrical dimethylarginine (ADMA) [8,9]. The association between elevated glucose and plasma ADMA was recently confirmed in human subjects [10]. Dysregulation of the ADMA catabolizing enzyme dimethylarginine dimethylaminohydrolase (DDAH) is thought to contribute to elevated systemic ADMA concentrations reported in type 2 diabetes, hypercholesterolemia and hypertension [11]. Thus, a possible mechanism leading to increased ADMA concentrations is reduced DDAH activity through high glucose [12]. The principal genes of ADMA metabolism are expressed in adipose tissue [13], but the physiological relevance has never been tested in animals or humans. We assume that an acute increase in plasma glucose concentrations or alterations in insulin sensitivity will induce changes in interstitial ADMA concentrations in humans. We validated clinical microdialysis (MD) to monitor interstitial ADMA concentrations in human adipose tissue, both in the fasting condition and during an oral glucose load. We studied subjects with expected differences in interstitial ADMA concentrations due to differences in body weight and glucose homeostasis (lean healthy, diabetic, and morbidly obese subjects before and after weight loss surgery). Circulating ADMA concentrations were also determined for comparison. A second aim was to assess adipose tissue DDAH activity in visceral and subcutaneous adipose tissue.

## 2. Results and Discussion

### 2.1. *In Vitro* Microdialysis Validation Experiments

**Figure 1.**
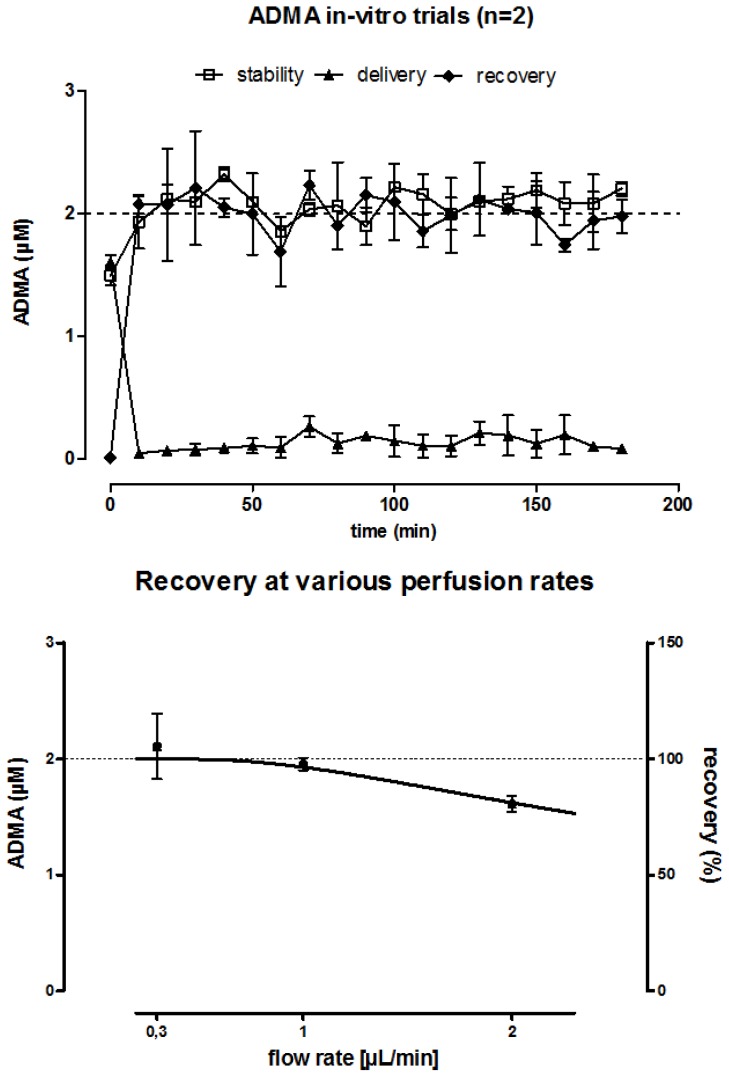
*In vitro* microdialysis. Stability, recovery and delivery experiments revealed stable asymmetrical dimethylarginine (ADMA) concentrations after approximately 20 min of catheter perfusion (upper panel, “equilibration period”). Recovery rate decreased with increasing flow rate (lower panel). Data are presented as mean and SEM of two independent experiments.

Using a custom-made *in vitro* MD system, we established and validated a sensitive and accurate technique to measure ADMA by GC-MS/MS in 1-μL aliquots of MD samples. By testing *in vitro* stability, recovery and delivery of ADMA, we determined a minimal equilibration period of 20 min before stable ADMA concentrations could be measured. With increasing flow rates from 0.3 to 2 μL/min, recovery rates decreased from 105% to 81% (Figure 1) as expected.

### 2.2. Clinical Microdialysis Experiments

Demographical data of the participating subjects are shown in Table 1.

**Figure 2.**
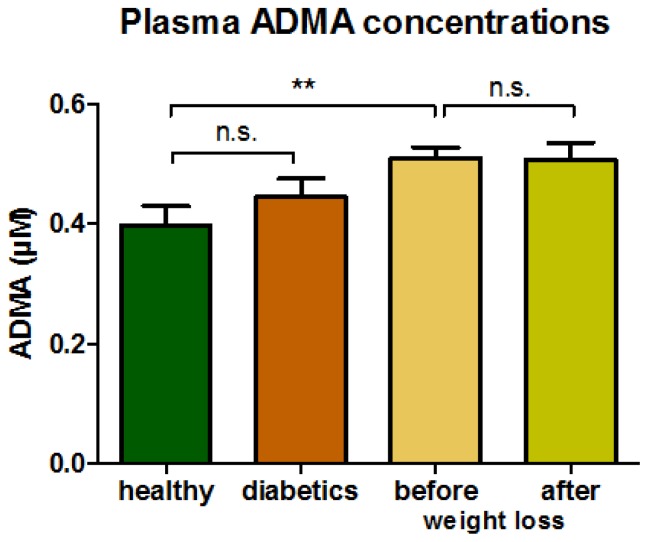
ADMA in plasma samples. Concentrations were 0.40 ± 0.03 μM in healthy subjects; 0.44 ± 0.03 μM in diabetics; 0.51 ± 0.02 μM before and 0.51 ± 0.03 μM after weight loss in morbidly obese subjects (** *p* < 0.01 healthy *vs.* morbidly obese; n.s.: not significant).

Circulating ADMA concentrations were lowest in normal weight healthy subjects and significantly higher in morbidly obese patients. ADMA concentrations did not change with weight loss, and were not different in diabetic patients compared to healthy controls (Figure 2).

Mean fasting interstitial adipose tissue ADMA concentrations, obtained as serial MD samples before glucose ingestion, did not differ between the different groups (lean healthy 0.17 ± 0.03 μM; diabetic 0.21 ± 0.03 μM; morbidly obese 0.16 ± 0.01 μM before surgery, and 0.17 ± 0.01 μM after weight loss). We did not observe any response of interstitial ADMA concentrations to the oral glucose challenge in the four groups of subjects.

**Figure 3.**
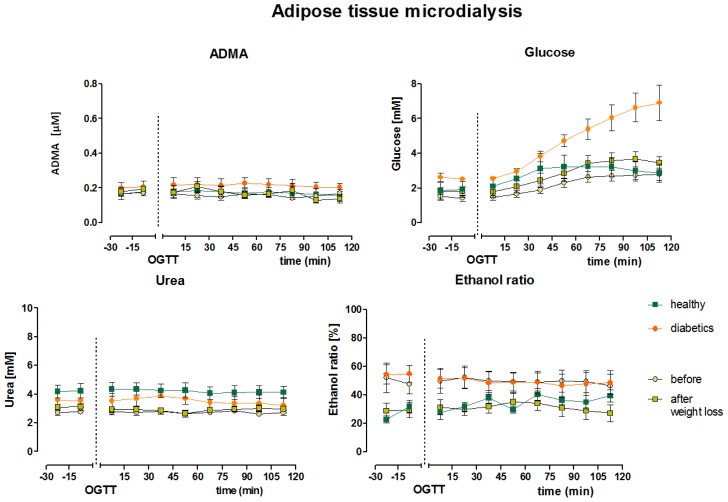
Clinical microdialysis (MD). Interstitial baseline ADMA values did not differ between the groups (lean healthy 0.17 ± 0.03 μM; diabetic 0.21 ± 0.03 μM; morbidly obese 0.16 ± 0.01 μM before surgery, and 0.17 ± 0.01 μM after weight loss). Interstitial glucose increased after oral glucose ingestion while ADMA, urea and ethanol ratio were not affected. (OGTT= oral glucose tolerance test).

Interstitial baseline glucose concentrations, obtained by MD at a flow rate of 1 μL/min, were within the expected range [14–16]. After oral glucose ingestion, interstitial glucose concentration slightly increased in all four groups (Figure 3) and was considerably higher in subjects with diabetes compared to healthy subjects (6.2 ± 1.0 *vs.* 2.9 ± 0.52 mmol/L, *p* < 0.05). Weight loss did not influence adipose tissue interstitial glucose concentrations in morbidly obese patients (2.5 ± 0.3 *vs.* 3.1 ± 0.4, mmol/L, *p* > 0.05). We determined changes in adipose tissue blood flow by the ethanol escape method (ethanol concentration in collected MD fluid/ethanol concentration in the original perfusion fluid) and urea recovery method. Both methods gave constant values throughout the experiments, indicating constant adipose tissue blood flow with the glucose load (Figure 3). Nevertheless, adipose tissue blood flow was higher in healthy lean subjects (ethanol ratio 27% ± 3%) compared to subjects with diabetes (ethanol ratio 50% ± 5%, *p* < 0.05, lower values indicating higher blood flow). Also, adipose tissue blood flow tended to increase with substantial weight loss (50% ± 9% and 31% ± 5% before and after weight loss, respectively, *p* = 0.059).

### 2.3. Adipose Tissue DDAH Activity

**Figure 4.**
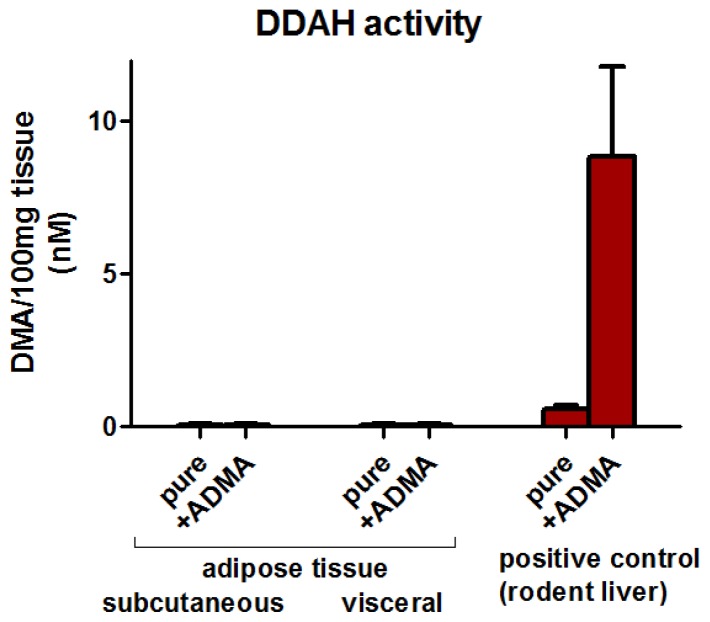
Adipose tissue dimethylarginine dimethylaminohydrolase (DDAH) activity. Subcutaneous and visceral human adipose tissue DDAH activity is negligible compared to DDAH activity in rodent liver. Data are given as mean and SEM (*n* = 5).

Subcutaneous and visceral adipose tissue DDAH activity was close to the lower limit of detection with no difference between the depots (subcutaneous adipose tissue: 0.2 ± 8.0 and visceral adipose tissue 1.9 ± 5.0 fmol DMA/min × mg). Compared to liver (8237 ± 2860 fmol DMA/min × mg), adipose tissue DDAH activity is negligible (Figure 4).

### 2.4. Discussion

A reliable analytical method based on serial MD sampling and GC-MS/MS was developed to continuously measure tissue interstitial ADMA levels in the clinical setting with a time resolution of 15 min over several hours. Time resolution could be even higher, given that we measured ADMA in 1-μL aliquots. Measurements of ADMA in human blood have long been established, but ADMA concentrations in the interstitial space of target tissues might provide more relevant information. Tissue interstitial ADMA concentrations had not previously been studied in humans. Our main finding is that interstitial adipose tissue ADMA concentrations are not different between morbidly obese subjects, subjects with diabetes, and normal weight healthy subjects, while plasma ADMA concentrations increase with higher BMI. Substantial weight loss, very surprisingly, did not change ADMA concentrations, neither in plasma nor interstitial values from adipose tissue. Another important finding is that rapid increases in plasma and interstitial glucose did not elicit significant increases in tissue ADMA concentrations, which is in line with a previous study in which no relation between food intake and ADMA levels could be established [17].

As indicated by previous studies, ADMA concentrations are closely related to the activity of ADMA metabolic enzymes (DDAH 1 and 2). Tissue insulin sensitivity is influenced by vascular glucose and insulin delivery to the interstitial space [18], whereas the endothelium regulates vascular homeostasis by the vasoactive mediator, NO, originated from NOS activity, which is in turn inhibited by ADMA [19]. In endothelial cell culture and tissue culture studies, even small changes in culture medium ADMA concentrations affected NOS activity [20]. Thus, a better understanding of local tissue production of ADMA *in situ* is important to elucidate the biological role of ADMA. We have developed a tool for such experiments.

Increased ADMA plasma concentrations were considered to be a major risk factor for type 2 diabetes and cardiovascular disease [19,21,22]. In a previous study, patients with the highest ADMA plasma concentrations had the lowest glucose tolerance [23]. Furthermore, elevated circulating ADMA in patients with insulin resistance was lowered by pharmacological improvement of glycemic control [8,24]. We did not observe such a strong relationship between interstitial glucose and ADMA concentrations in healthy obese subjects and subjects with diabetes. Furthermore, a strong relationship between BMI and plasma ADMA [25], improved endothelial function after weight loss [1,2,26], and decreased circulating ADMA after weight loss [27,28] were all described in previous studies. In our study, lower tissue blood flow was observed in diabetic patients compared to healthy normal weight control subjects and, after weight reduction, adipose tissue blood flow tended to increase. The observed higher tissue blood flow might be an indication for improved endothelial function which would be in line with previous studies [29–31]. Despite these findings, interstitial ADMA concentrations in our group of morbidly obese patients did not differ after a mean weight loss of 34 ± 13 kg. A possible explanation for the conflicting findings might be a different microdialysis recovery rate, because recovery depends on local blood flow [32]. Admittedly, the study has been carried out on small groups of subjects; therefore the observed difference in blood flow between our tested groups might have concealed the changes in local ADMA concentrations in adipose tissue. A possible mechanism by which endothelial function is altered may be due to up-regulation of DDAH. In rats, pioglitazone increased NO production partly by up-regulating tissue DDAH expression [33]. We measured DDAH activity both in subcutaneous and visceral adipose tissue. The enzyme activity was negligible compared to liver tissue which possesses high DDAH activity [34]. Considering our present findings, ADMA in adipose tissue seems to play a minor role in NO-dependent regulation of adipose tissue blood flow and metabolism. In human adipose tissue, gene expression of all enzymatic components of ADMA metabolism have been identified [13], but obviously the gene expression levels do not reflect DDAH activity. Overall, the role of local ADMA production in adipose tissue remains to be elucidated, but our findings at least suggest a minor role for blood flow regulation.

## 3. Experimental Section

### 3.1. Study Subjects

We enrolled four patients with diabetes mellitus, four healthy control subjects, and seven morbidly obese patients before and after substantial weight loss (mean weight loss 34 ± 13 kg at least 6 month after bariatric surgery). In these subjects we performed subcutaneous adipose tissue microdialysis to evaluate changes in interstitial ADMA concentrations throughout oral glucose tolerance testing (75 g glucose in 300 mL water). All participants were non-smokers and had no history of gastrointestinal, endocrinological, or psychiatric disorders, and presented with normal physical exams and electrocardiograms. Blood pressure was increased I some participants (see Table 1). Blood tests confirmed normal blood count and normal liver and renal function. Adipose subcutaneous and visceral tissue samples were obtained from five obese patients undergoing laparoscopic bariatric surgery. All subjects taking part provided written informed consent to these studies which have been approved by the Hannover Medical School Ethics Committee in Hannover, Germany.

### 3.2. Microdialysis

We employed a custom-made *in vitro* microdialysis system [35] to evaluate ADMA *in vitro* stability, delivery, and recovery using a clinical MD catheter (CMA 60, CMA/μDialysis, Stockholm, Sweden), and lactate-free Ringer solution (B. Braun, Melsungen, Germany). The *in vitro* data were employed to determine the best conditions for *in vivo* microdialysis.

For clinical microdialysis, all subjects were studied after an overnight fast while resting in the supine position on a comfortable bed in a room kept at 23 °C. Microdialysis catheters were inserted under sterile conditions into adipose tissue (8 to 10 cm left of the umbilicus) and connected to a microdialysis pump (CMA 107, CMA Microdialysis AB, Stockholm, Sweden). After probe insertion, catheters were perfused at a flow rate of 1 μL/min with sterile Ringer solution containing 50 mM ethanol. Following a sufficient equilibration period of at least 30 min, microdialysis samples were collected every 15 min for 30 min before and up to two hours after oral glucose ingestion. Microdialysis samples were placed on ice immediately after collection and stored at −80° C until analysis.

### 3.3. Adipose Tissue Collection

About 1–5 g adipose tissue were dissected from each depot during regular endoscopic surgery. Adipose tissue pieces were immediately frozen on dry ice and stored at −80 °C until further analysis.

### 3.4. Analytical Procedures

ADMA and its precursor were measured simultaneously in plasma and in 1-μL aliquots of MD samples by a specific and sensitive GC-MS/MS (gas chromatography-tandem mass spectrometry) method [36]. Quantification was performed by selected-reaction monitoring of the mass transition *m/z* 634 → *m/z* 378 for ADMA and *m/z* 637 → *m/z* 378 for the internal standard trideuteromethyl-ADMA in the negative-ion chemical ionization mode. Besides ADMA, *in vivo* MD samples were analyzed for glucose and urea concentrations using the automated enzyme-linked spectrophotometric CMA 600 analyzer (CMA, Solna, Sweden) [37]. Ethanol concentration was measured using the standard enzymatic assay with alcohol dehydrogenase (Sigma-Aldrich, Seelze, Germany) and a plate reader (Tecan, Infinite F200, Männedorf, Switzerland) as described before [38]. Changes in local blood flow were determined using both the ethanol escape technique, and by urea clearance [39,40].

DDAH activity was assessed in adipose tissue and, as a positive control, in mice liver tissue. A GC-MS (gas chromatography-mass spectrometry) assay was applied as described previously [34]. The method is based on the quantitative determination of ADMA-derived dimethylamine (DMA). Quantification was performed by selected-ion monitoring of the ions *m/z* 240 for DMA and *m/z* 246 for the internal standard hexadeutero-DMA in the negative-ion chemical ionization mode.

### 3.5. Statistical Analysis

Data are shown as mean and standard error. Group comparison was done with Students *t*-test for paired or unpaired samples, as appropriate. *p* < 0.05 was considered to be statistically significant.

## 4. Conclusions

We have adapted a sensitive analytical method to measure tissue interstitial ADMA concentrations in microdialysis samples in a clinical setting. In our study, carried out in a limited number of subjects, acute changes in tissue ADMA concentrations following glucose intake were not observed. DDAH activity, both in subcutaneous and visceral adipose tissue, was extremely low compared to liver tissue. Further studies are warranted to establish the role of interstitial ADMA for the regulation of adipose tissue metabolism. However, we believe that the suggestion of ADMA playing a major role in adipose blood flow regulation can be rejected.

## Figures and Tables

**Table 1.** Demographical data of the participating subjects.

	Healthy subjects (*n* = 4)	Patients with diabetes (*n* = 4)	Patients with morbid obesity before weight loss (*n* = 7) *	Patients after weight loss (*n* = 7) *
Age (year)	38 ± 4	54 ± 8	35 ± 3	36 ± 3
Sex (m/f)	4/0	1/3	2/5	2/5
BMI (kg/m^2^)	23.9 ± 1.8	30.0 ± 5.2	45.7 ± 1.5	34.4 ± 2.0
Weight (kg)	77 ± 2	80 ± 1	135 ± 4	102 ± 5
HOMA	1.2 ± 0.3	3.7 ± 0.3	5.6 ± 2.0	1.8 ± 0.7
HbA1c (%)	<6.5	7.6 ± 1.3	<6.5	<6.5
Hypertension (*n*)	0	3	4	0
Other comorbidities (*n*)	0	allergy (1)	migraine (2)	migraine (2)

Drug treatment (*n*)	none	ACE-inhibitors (2); beta blockers (1) thiazide diuretics (1) metformin (3) insulin (1)	AT1-blockers (1) ACE-inhibitors (1) diuretics (1) beta blockers (3) topiramate (1) diclofenac (1) omeprazole (3)	topiramate (1) diclofenac (1) omeprazole (1)

* paired samples.

## References

[1] Ziccardi P., Nappo F., Giugliano G., Esposito K., Marfella R., Cioffi M., D’Andrea F., Molinari A.M., Giugliano D. (2002). Reduction of inflammatory cytokine concentrations and improvement of endothelial functions in obese women after weight loss over one year. Circulation.

[2] Hamdy O., Ledbury S., Mullooly C., Jarema C., Porter S., Ovalle K., Moussa A., Caselli A., Caballero A.E., Economides P.A. (2003). Lifestyle modification improves endothelial function in obese subjects with the insulin resistance syndrome. Diabetes Care.

[3] Pi-Sunyer F.X. (2002). The obesity epidemic: Pathophysiology and consequences of obesity. Obes. Res.

[4] Caballero A.E. (2003). Endothelial dysfunction in obesity and insulin resistance: a road to diabetes and heart disease. Obes. Res.

[5] Mohamed-Ali V., Pinkney J.H., Coppack S.W. (1998). Adipose tissue as an endocrine and paracrine organ. Int. J. Obes. Relat. Metab. Disord.

[6] Bergman R.N., Mittelman S.D. (1998). Central role of the adipocyte in insulin resistance. J. Basic Clin. Physiol. Pharmacol.

[7] Yudkin J.S., Eringa E., Stehouwer C.D.A. (2005). “Vasocrine” signalling from perivascular fat: A mechanism linking insulin resistance to vascular disease. Lancet.

[8] Stuhlinger M.C., Abbasi F., Chu J.W., Lamendola C., McLaughlin T.L., Cooke J.P., Reaven G.M., Tsao P.S. (2002). Relationship between insulin resistance and an endogenous nitric oxide synthase inhibitor. JAMA.

[9] Cooke J.P. (2000). Does ADMA cause endothelial dysfunction?. Arterioscler. Thromb. Vasc. Biol.

[10] Konukoglu D., Firtina S., Serin O. (2008). The relationship between plasma asymmetrical dimethyl-L-arginine and inflammation and adhesion molecule levels in subjects with normal, impaired, and diabetic glucose tolerance. Metabolism.

[11] Tran C.T., Leiper J.M., Vallance P. (2003). The DDAH/ADMA/NOS pathway. Atheroscler. Suppl.

[12] Lin K.Y., Ito A., Asagami T., Tsao P.S., Adimoolam S., Kimoto M., Tsuji H., Reaven G.M., Cooke J.P. (2002). Impaired nitric oxide synthase pathway in diabetes mellitus: Role of asymmetric dimethylarginine and dimethylarginine dimethylaminohydrolase. Circulation.

[13] Spoto B., Parlongo R.M., Parlongo G., Sgro’ E., Zoccali C. (2007). The enzymatic machinery for ADMA synthesis and degradation is fully expressed in human adipocytes. J. Nephrol.

[14] Ekberg N.R., Wisniewski N., Brismar K., Ungerstedt U. (2005). Measurement of glucose and metabolites in subcutaneous adipose tissue during hyperglycemia with microdialysis at various perfusion flow rates. Clin. Chim. Acta.

[15] Strindberg L., Lonnroth P. (2000). Validation of an endogenous reference technique for the calibration of microdialysis catheters. Scand. J. Clin. Lab. Invest.

[16] Reinstrup P., Stahl N., Mellergard P., Uski T., Ungerstedt U., Nordstrom C.H. (2000). Intracerebral microdialysis in clinical practice: Baseline values for chemical markers during wakefulness, anesthesia, and neurosurgery. Neurosurgery.

[17] Engeli S., Tsikas D., Lehmann A.C., Böhnke J., Haas V., Strauß A., Janke J., Gorzelniak K., Luft F.C., Jordan J. (2012). Influence of dietary fat ingestion on asymmetrical dimethylarginine in lean and obese human subjects. Nutr. Metab. Cardiovasc. Dis.

[18] Wasserman D.H. (2009). Four grams of glucose. Am. J. Physiol. Endocrinol. Metab.

[19] Boger R.H., Vallance P., Cooke J.P. (2003). Asymmetric dimethylarginine (ADMA): A key regulator of nitric oxide synthase. Atheroscler. Suppl.

[20] Cardounel A.J., Cui H., Samouilov A., Johnson W., Kearns P., Tsai A.L., Berka V., Zweier J.L. (2007). Evidence for the pathophysiological role of endogenous methylarginines in regulation of endothelial NO production and vascular function. J. Biol. Chem.

[21] Vallance P., Leone A., Calver A., Collier J., Moncada S. (1992). Accumulation of an endogenous inhibitor of nitric oxide synthesis in chronic renal failure. Lancet.

[22] Boger R.H. (2003). The emerging role of asymmetric dimethylarginine as a novel cardiovascular risk factor. Cardiovasc. Res.

[23] Miyazaki H., Matsuoka H., Cooke J.P., Usui M., Ueda S., Okuda S., Imaizumi T. (1999). Endogenous nitric oxide synthase inhibitor: A novel marker of atherosclerosis. Circulation.

[24] Asagami T., Abbasi F., Stuelinger M., Lamendola C., McLaughlin T., Cooke J.P., Reaven G.M., Tsao P.S. (2002). Metformin treatment lowers asymmetric dimethylarginine concentrations in patients with type 2 diabetes. Metabolism.

[25] Eid H.M., Arnesen H., Hjerkinn E.M., Lyberg T., Seljeflot I. (2004). Relationship between obesity, smoking, and the endogenous nitric oxide synthase inhibitor, asymmetric dimethylarginine. Metabolism.

[26] Raitakari M., Ilvonen T., Ahotupa M., Lehtimaki T., Harmoinen A., Suominen P., Elo J., Hartiala J., Raitakari O.T. (2004). Weight reduction with very-low-caloric diet and endothelial function in overweight adults: role of plasma glucose. Arterioscler. Thromb. Vasc. Biol.

[27] Krzyzanowska K., Mittermayer F., Kopp H.P., Wolzt M., Schernthaner G. (2004). Weight loss reduces circulating asymmetrical dimethylarginine concentrations in morbidly obese women. J. Clin. Endocrinol. Metab.

[28] McLaughlin T., Stuhlinger M., Lamendola C., Abbasi F., Bialek J., Reaven G.M., Reaven G.M., Tsao P.S. (2006). Plasma asymmetric dimethylarginine concentrations are elevated in obese insulin-resistant women and fall with weight loss. J. Clin. Endocrinol. Metab.

[29] Anderson T.J., Uehata A., Gerhard M.D., Meredith I.T., Knab S., Delagrange D., Lieberman E.H., Ganz P., Creager M.A., Yeung A.C. (1995). Close relation of endothelial function in the human coronary and peripheral circulations. J. Am. Coll. Cardiol.

[30] Williams S.B., Goldfine A.B., Timimi F.K., Ting H.H., Roddy M.A., Simonson D.C., Creager M.A. (1998). Acute hyperglycemia attenuates endothelium-dependent vasodilation in humans *in vivo*. Circulation.

[31] Jansson P.A. (2007). Endothelial dysfunction in insulin resistance and type 2 diabetes. J. Intern. Med.

[32] Plock N., Kloft C. (2005). Microdialysis—Theoretical background and recent implementation in applied life-sciences. Eur. J. Pharm. Sci.

[33] Wakino S., Hayashi K., Tatematsu S., Hasegawa K., Takamatsu I., Kanda T., Homma K., Yoshioka K., Sugano N., Saruta T. (2005). Pioglitazone lowers systemic asymmetric dimethylarginine by inducing dimethylarginine dimethylaminohydrolase in rats. Hypertens. Res.

[34] Chobanyan K., Thum T., Suchy M.T., Zhu B., Mitschke A., Gutzki F.M., Beckmann B., Stichtenoth D.O., Tsikas D. (2007). GC-MS assay for hepatic DDAH activity in diabetic and non-diabetic rats by measuring dimethylamine (DMA) formed from asymmetric dimethylarginine (ADMA): Evaluation of the importance of *S*-nitrosothiols as inhibitors of DDAH activity *in vitro* and *in vivo* in humans. J. Chromatogr. B.

[35] May M., Batkai S., Zoerner A.A., Tsikas D., Jordan J., Engeli S. (2013). Enhanced human tissue microdialysis using hydroxypropyl-ss-cyclodextrin as molecular carrier. PLoS One.

[36] Tsikas D., Schubert B., Gutzki F.M., Sandmann J., Frolich J.C. (2003). Quantitative determination of circulating and urinary asymmetric dimethylarginine (ADMA) in humans by gas chromatography-tandem mass spectrometry as methyl ester tri(*N*-pentafluoropropionyl) derivative. J. Chromatogr. B.

[37] Tholance Y., Barcelos G., Quadrio I., Renaud B., Dailler F., Perret-Liaudet A. (2010). Analytical validation of microdialysis analyzer for monitoring glucose, lactate and pyruvate in cerebral microdialysates. Clin. Chim. Acta.

[38] Adams F., Jordan J., Schaller K., Luft F.C., Boschmann M. (2005). Blood flow in subcutaneous adipose tissue depends on skin-fold thickness. Horm. Metab. Res.

[39] Fellander G., Linde B., Bolinder J. (1996). Evaluation of the microdialysis ethanol technique for monitoring of subcutaneous adipose tissue blood flow in humans. Int. J. Obes. Relat. Metab. Disord.

[40] Farnebo S., Zettersten E.K., Samuelsson A., Tesselaar E., Sjoberg F. (2011). Assessment of blood flow changes in human skin by microdialysis urea clearance. Microcirculation.

